# A doxycycline-inducible urokinase receptor (uPAR) upregulates uPAR activities including resistance to anoikis in human prostate cancer cell lines

**DOI:** 10.1186/1476-4598-6-34

**Published:** 2007-05-17

**Authors:** Mohammad Hasanuzzaman, Robert Kutner, Siamak Agha-Mohammadi, Jakob Reiser, Inder Sehgal

**Affiliations:** 1 LSU Department of Comparative Biomedical Sciences, Louisiana State University, Baton Rouge, LA 70803, USA; 2 LSU Health Sciences Center New Orleans, Gene Therapy Program, Department of Medicine, New Orleans, LA 70112, USA; 3 University of Pittsburgh Medical Center, Pittsburgh, PA 15261, USA

## Abstract

**Background:**

The urokinase receptor (uPAR) mediates a diverse array of cellular processes including several events involved in prostate cancer metastasis. Many of these activities are initiated or enhanced by uPAR binding to its proteolytic ligand, urokinase (uPA). Our objective in this study was to generate and test an inducible lentiviral system capable of expressing uPAR and DsRed fluorescent protein in human prostate cancer cell lines.

**Results:**

A DsRed-uPAR fusion construct was inserted into a lentiviral vector. Transduction of human prostate cancer cell lines with this virus and with a virus containing a reverse-tetracycline transactivator (rt-TA) resulted in a stable transgene which induced both uPAR and DsRed proteins in a dose-responsive fashion upon stimulation with doxycycline. Immunoblots and immunofluorescence studies indicated no detectable uPAR expression in non-induced prostate cancer cell lines. Cells with induced-uPAR demonstrated increased cellular adhesion to the matrix substrate vitronectin and increased net cell proliferation compared to uninduced cells. Finally, induced uPAR-expressing prostate cancer cells were resistant to anoikis over an extended time period when grown in suspension.

**Conclusion:**

This doxycycline-inducible lentivirus system produces titerable levels of biologically active uPAR *in vitro*. This tool can be used to dissect cellular events following induction of uPAR in prostate cancer cells.

## Background

The urokinase receptor (uPAR) contributes to a diverse array of cellular processes such as intravascular homeostasis, inflammation, cancer metastasis, proliferation, activation of protease cascades, matrix degradation and cell migration. Many but not all of these activities are initiated or enhanced by uPAR binding to its proteolytic ligand, urokinase (uPA) [1,2]. Molecularly, uPAR consists of 3 separate domains forming a bowl which binds uPA within the central cavity [3]. Recently, a 2.8 Å resolution crystal structure of a soluble uPAR fragment was described and this structure demonstrates that residues within all three domains participate in uPA binding. This crystal structure also suggests how uPA binding may induce substantial conformational changes in uPAR to reposition receptor subdomains and increase the affinity and specificity of these subdomains towards pericellular proteins such as vitronectin, various integrins, low-density lipoprotein receptor, epidermal growth factor receptor and other receptors [3].

*In vitro *and *in vivo *models to study uPAR biology are improving and with these models are coming novel identifications of cellular processes upregulated by uPAR action. We have recently found that in an *in vivo *assay of orthotropic metastasis to regional lymph nodes, metastases which succeed in invading as far as these lymph nodes and then proliferated within the nodes possessed significantly greater numbers of uPAR-positive staining cells when compared to the general population of cells within the primary tumor [4]. To investigate how increased uPAR might promote tumor metastasis, we modeled metastatic transit by placing cells in suspension culture. Although this suspension normally induces a form of apoptosis called "anoikis", we found that suspension resulted in an increase in uPAR cell surface expression. Using transient transfection, we reported that cells showing high uPAR appeared more resistant to anoikis than lower expressing cells [4].

Transfection of uPAR produced high receptor levels in a relatively low percent prostate cancer cells and thus we wished to greatly enhance the percentage of uPAR-overexpressing cells in follow-up experiments. In addition, the transient nature of transfected plasmid expression limited the potential time course of anoikis assays. Therefore we designed a regulatable approach to control transgene expression of uPAR utilizing a tetracycline (tet)-inducible vector. Tetracycline-regulated gene expression is the most common and well characterized method of controlling transgene expression. It allows gene induction following administration of a tetracycline antibiotic such as doxycycline. Binding of doxycycline to a series of tet resistance operon sequences transactivates a minimal CMV promoter activating gene expression in the presence of antibiotic [5,6]. Unfortunately, inducible systems have been traditionally plagued by weakness of leak-through of unwanted gene expression or inadequate inducibility [7]. To circumvent these potential problems, we have used a recently redesigned tet-inducible lentivirus vector system. This lentivirus vector possesses a chicken β-globin insulator sequence to insulate the minimal promoter from chromosome position effects and a second-generation tetracycline response element (TRE) with optimized distance and rotation between tet operator (tetO) sequences. In addition, many of the U3 proviral long terminal repeat sequences have been deleted [7].

In this study, we report the development and employment of this inducible model system to trigger controlled uPAR induction by treatment with doxycycline. This model has no detectable leakage of uPAR expression in non-induced cells and produces the co-expression of the fluorescent DsRed protein in doxycycline-stimulated cells. We have employed this lentiviral system in two prostate cancer cell lines which express low or no detectable uPAR. We show the dose-responsive induction of both uPAR and DsRed following doxycycline administration. Using the cells generated with this lentiviral system, we have assessed a number of biological responses associated with increased uPAR such as adhesion to vitronectin and proliferation. We also used the inducible cells along with controls to further substantiate the ability of uPAR to provide significant resistance to anoikis in these cells lacking of wild-type uPAR.

## Results

### A culture variant of the PC-3M cell line expresses no detectable uPAR protein

**Figure 1:**
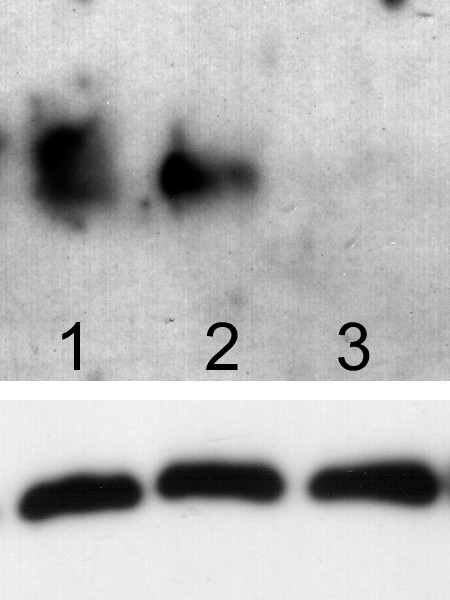
Upper panel shows expression of endogenous uPAR protein expression in three PC-3 derived human prostate cancer cell lines. Lower panel shows β-actin loading control. 1-PC-3M cells; 2-PC-3 cells, 3-PC-3M-CBS cells.

A variant PC-3M cell line which expressed little or no detectable uPAR when compared with either parental PC-3M cells or PC-3 cells developed spontaneously in culture (Figure 1A). We selected this cell variant (which we named "PC-3M-CBS") and the LNCaP prostate cancer line to study the effect of inducing uPAR against a background of little or no endogenous protein expression.

### Doxycycline induces uPAR and DsRed protein expression in a dose-responsive pattern in two prostate cancer cell lines

**Figure 2:**
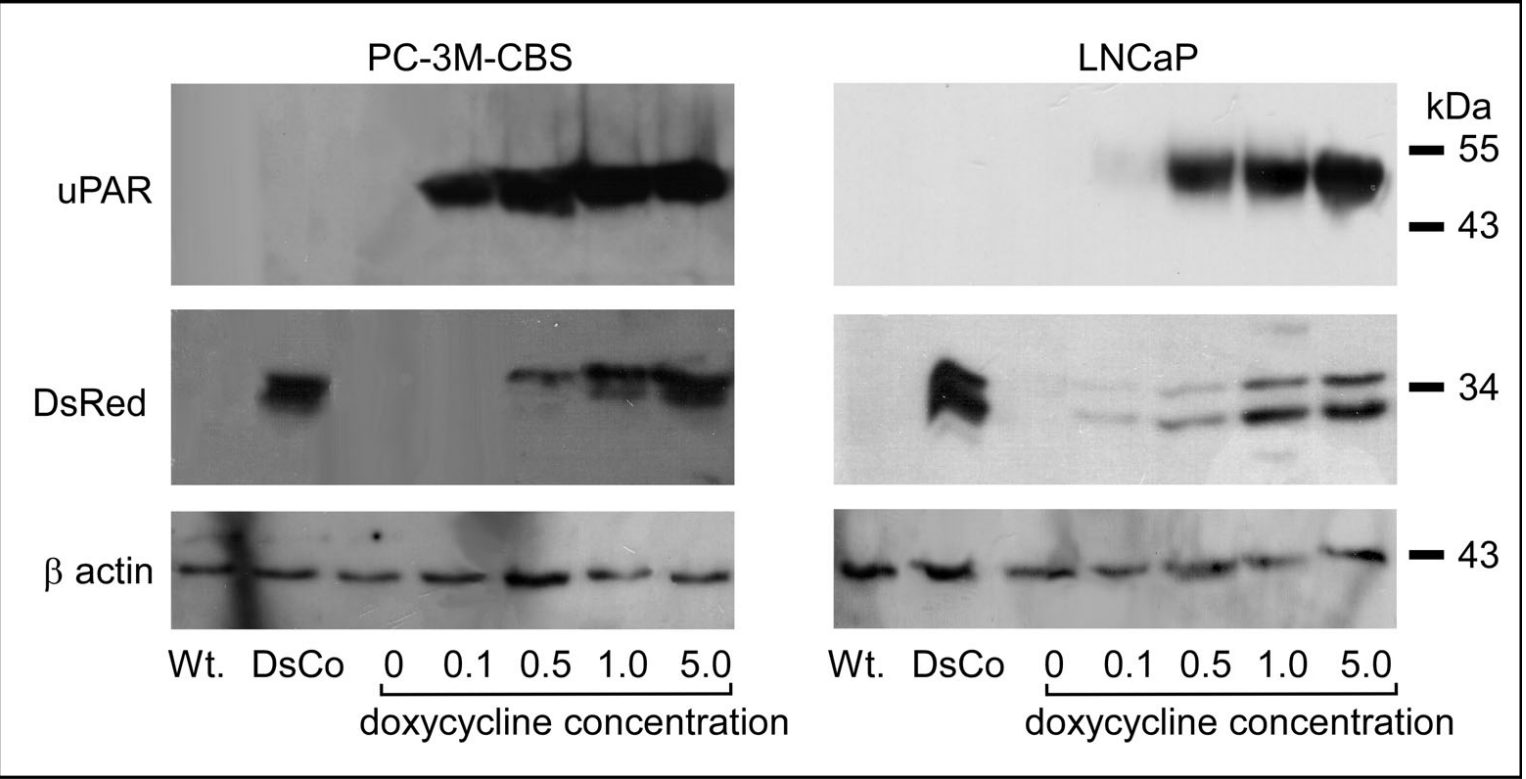
Induction of uPAR and DsRed protein expression in human prostate cancer cells. Fifty micrograms of protein lysate was applied to each lane. Upper panels shows uPAR expression in wild-type PC-3M-CBS and LNCaP cells (Wt.) and in cells transduced with DsRed virus (DsCo; as a control for potential effects due to DsRed expression), relative to levels in cells expressing doxycycline-inducible uPAR. uPAR-DsRed was induced in these cells by stimulation with doxycycline HCL at the indicated concentrations for 4 days prior to lysis and immunoblot. Middle panel shows DsRed monomer expression in wild-type, DsRed control and doxycycline-induced cells. Lower panel shows beta-actin expression as a control for loading.

PC-3M-CBS and LNCaP cells were transduced with an inducible lentiviral construct bearing a DsRed-uPAR cDNA fusion. Since uPAR is post-translationally processed by removal of amino acids from both the amino and carboxy terminals, uPAR and DsRed proteins are expressed separately and therefore migrate according to their true molecular mass. In PC-3M-CBS cells, doxycycline induced robust uPAR expression at 0.1 ug/mL, this expression increased with greater levels of doxycycline and appeared to reach a plateau at 1.0 ug/mL (Figure 2). No uPAR signal appeared in either the uninduced, wild-type or DsRed expressing cells when loaded at 50 ug total protein per electrophoresis lane. DsRed protein in these cells also increased in a dose-responsive fashion after doxycycline stimulation but appreciable levels of DsRed monomer appeared only after 0.5 ug/mL treatment. The slight variance between observed uPAR and DsRed protein levels in these immunoblots may reflect differences in the half-lives of uPAR and DsRed monomer in these cells. DsRed protein appeared as two bands with the more prominent band being a higher molecular weight. These two bands likely indicate that there are at least two sites of post-translational cleavage of DsRed from uPAR.

LNCaP prostate cancer cells were also transduced with the inducible lentiviral vector. These cells also showed a dose-response to doxycycline stimulation with regard to uPAR induction; however a 0.5 ug/mL concentration was required to induce high levels of protein expression. In these cells, the uPAR signal also appeared to plateau at the 1.0 ug/mL concentration. DsRed expression was increased by increasing doxycycline in the LNCaP line and again, the monomer appeared in two distinct bands. Because these results indicated that uPAR expression was maximal after 1.0 ug/mL doxycycline and because this level of stimulation also produced substantial levels of DsRed monomer, we used 1.0 ug/mL doxycycline throughout subsequent experiments.

**Figure 3:**
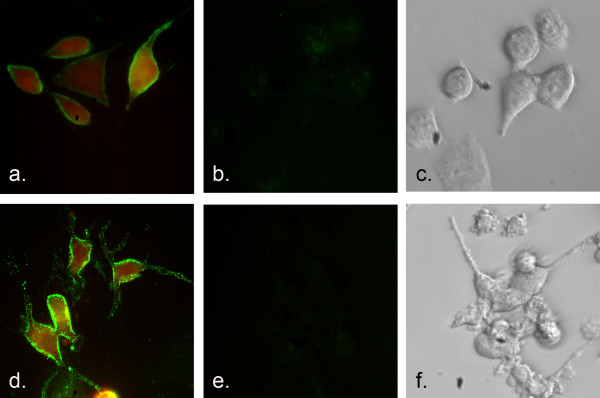
uPAR and DsRed expression in prostate cancer cells (200 × images). PC-3M-CBS cells containing uPAR-DsRed lentivirus were induced with doxycycline over a 4-day period. The overlay image (a) shows expression of both uPAR (green fluorescence) and DsRed. Uninduced cells (b) show no fluorescence. Phase contrast image (c) of uninduced cells depict cells present in (b). LNCaP cells transduced with the lentivirus show a similar positive (d) and negative expression patterns (e & f).

**Figure 4:**
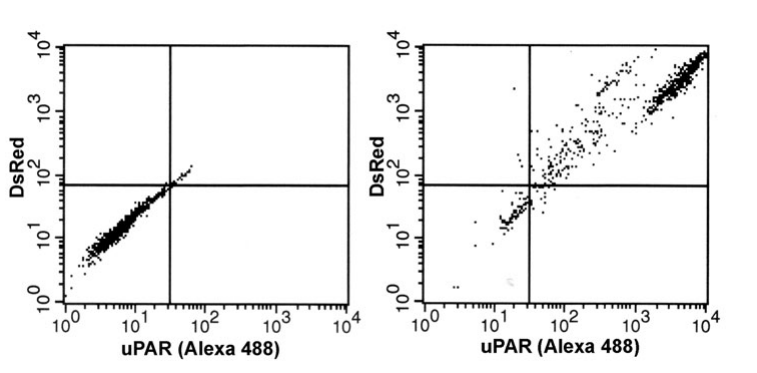
Flow cytometry of uPAR cell surface expression and DsRed fluorescence. Representative scatter plot of uPAR immuno-fluorescence (x-axis) and DsRed fluorescence (y-axis) by lentivirus-transduced PC-3M-CBS cells. Left panel shows fluorescence scatter in uninduced cells; right panel shows fluorescence scatter in doxycycline-induced cells. uPAR was detected with AlexaFluor 488 secondary goat anti-mouse antibody.

Immunoreactive expression of uPAR and fluorescence from DsRed protein followed stimulation with 1.0 ug/mL doxycycline. These induced cells displayed pronounced levels of both proteins compared with uninduced cells (Figure 3). Post-translational cleavage of DsRed from uPAR results in separate proteins and in these images, DsRed protein is distributed cytoplasmically while uPAR is seen at the cell surface. A representative flow cytometry scatter plot shows the shift in fluorescence after overall induction of both proteins in the cell population (Figure 4).

### uPAR induction or supplemental uPA addition promote increased proliferation in PC-3M-CBS and LNCaP cells

**Figure 5:**
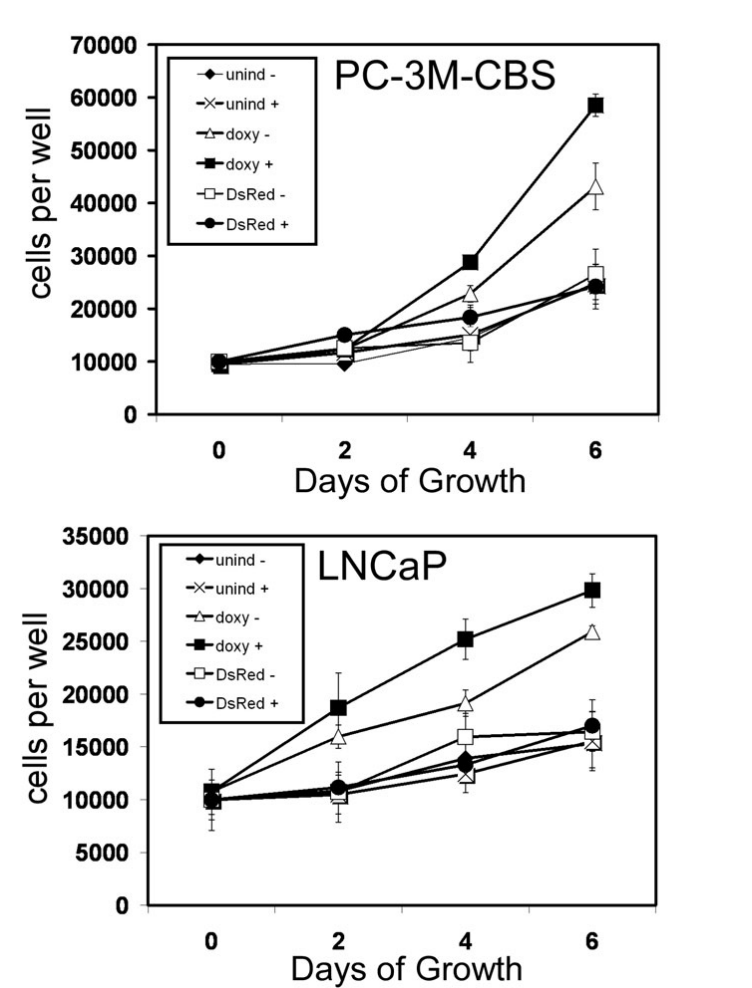
Effect of induced uPAR expression on proliferation. Prostate cancer cells were either uninduced or induced to express uPAR-DsRed with 1.0 ug/mL doxycycline. DsRed-expressing cells were used as a growth control. Cells were plated at 10,000 cells per well in 24-well plates and grown in culture medium containing 2% fetal bovine serum with (+) or without urokinase (uPA; 100 pM). Medium was changed during the third day. Symbols and indicated conditions are: ◆ uninduced, without uPA; × uninduced plus uPA; △ doxycycline induced without uPA; ■ doxycycline induced plus uPA; □ DsRed control without uPA; ● DsRed control plus uPA. Data reflects the means of 4 wells. Bars, SE.

We have previously found that uPA can stimulate growth of prostate cancer cells over a range of concentrations and that maximal growth stimulation in a cell line which expresses relatively high levels of uPAR, occurred at 100 pM [8]. To understand the potential effect of uPA on proliferation in uPAR-inducible cells, we therefore added 100 pM uPA to doxycycline-induced or uninduced cultures and measured increases in cell number over a 6-day period. DsRed expressing cell lines were used for comparison to ascertain the effect of DsRed expression on growth rate. In PC-3M-CBS cultures, this experiment revealed that uPAR induction without addition of uPA promoted an approximately 2-fold increase in proliferation compared with uninduced cells or DsRed controls without added uPA (Figure 5A). However, addition of uPA to uPAR-induced cells further stimulated growth. uPA addition to either DsRed controls or uninduced cells did not alter growth.

Uninduced LNCaP cells treated with uPA grew equivalently to uninduced cells without uPA and to DsRed control cells with or with out uPA indicating that uPA did not effect proliferation in this cell line (Figure 5B). These results are consistent with the documented lack of uPAR in the LNCaP cell line [9]. When uPAR was induced with doxycycline however, growth was increased. Addition of uPA further increased LNCaP growth during the 6 day period. These results indicate that induced expression of the uPAR-DsRed fusion is functional and can couple with existing signal transduction machinery to allow uPA-mediated proliferation.

### uPAR expression enhances cellular adhesion to vitronectin

**Figure 6:**
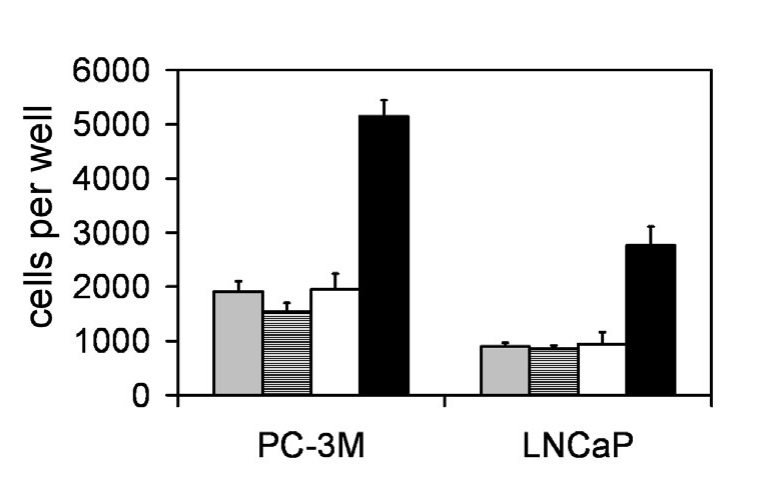
Effect of induced uPAR expression on adhesion to vitronectin. Adhesion of prostate cancer cells to vitronectin is increased by induction of uPAR by doxycycline. Prostate cancer cells from wild-type control cultures (grey bars), DsRed-expressing controls (striped bars), un-induced cultures (white bars) and uPAR-induced cells (black bars) were plated into vitronectin-coated wells in a 24-well plate. Following a one-hour period of attachment, the cells were detached and counted as described in Methods. Data is expressed as the number of adherent cells per well; error bars show the SD from quadruplicate wells.

Wild-type and DsRed-expressing control cells from both PC-3M-CBS and LNCaP adhered to a vitronectin-coated surface in equivalent numbers as compared to the respective cells which were transduced with uPAR-DsRed but were un-induced (Figure 6). The LNCaP line had much weaker constitutive adhesion to the vitronectin surface. Doxycycline induction of uPAR in both prostate cancer cell lines however, lead to a pronounced increase in the level of attachment indicating that induced uPAR is biologically functional in mediating vitronectin matrix adhesion.

### uPAR induction provided substantial resistance to anoikis

**Figure 7:**
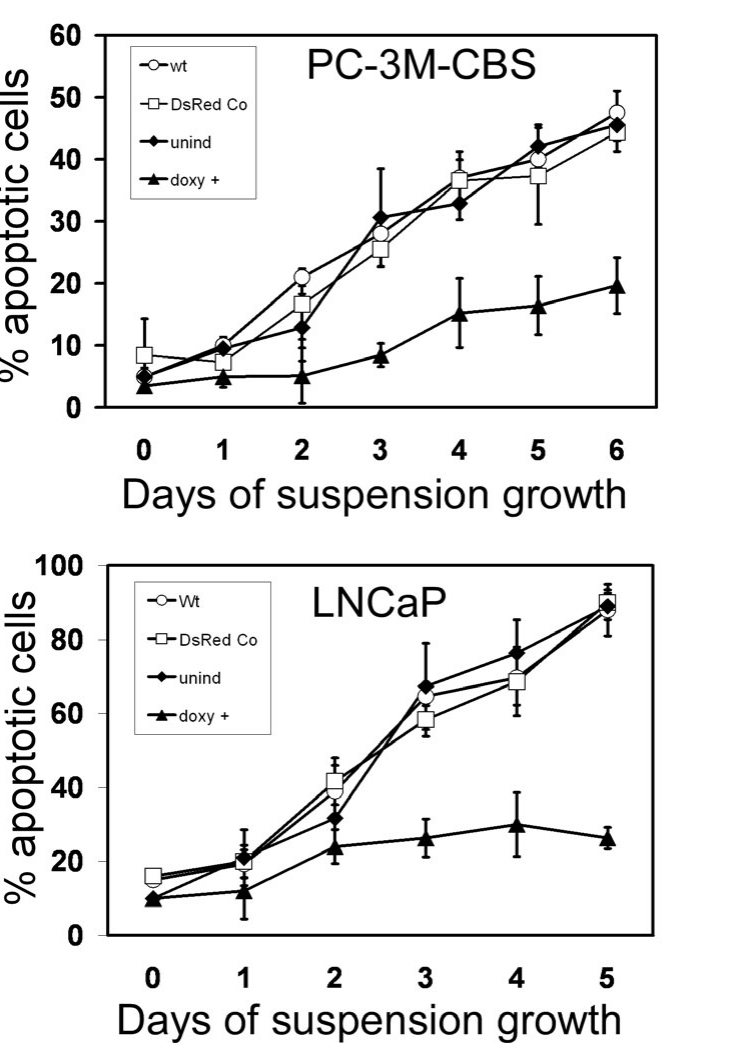
Quantitation of Anoikis. The percentage of apoptosis in suspended cells (anoikis) was quantitated over time. Apoptosis was calculated from cells which stained positively for annexin V. Live cells were calculated as those which stained positively for Calcein blue methoxyester and did not stain with annexin V. Symbols and indicated conditions are: ○ Wild-type cells; □ DsRed control cells; ◆ uninduced cells containing uPAR-DsRed virus; ▲ doxycycline-induced cells containing uPAR-DsRed virus. Data represent mean ± S.D. of nine regions counted per slide per day.

To model the state of detachment in which cancer cells must exist during metastasis, both cancer cell lines were placed in forced suspension culture and incubated in suspension over a period of days during which the numbers of early apoptotic vs. live cells were quantitatively scored. This experiment demonstrated that cells with induced uPAR were significantly more resistant to developing anoikis during this long term incubation in suspension (Figure 7). This anoikis resistance was more dramatic in the LNCaP line than in the PC-3M-CBS cells. In LNCaP cells, uninduced, wild-type or DsRed control cells reached 90% annexin positive staining at 5 days, a level more than 3-fold greater than uPAR-induced LNCaP cells. Compared with uninduced PC-3M-CBS cells, the greater apoptotic levels in uninduced LNCaP likely reflect greater general sensitivity towards anoikis.

## Discussion

In this study, we describe the construction of an inducible system to model uPAR overexpression in prostate cancer cells. The use of lentivirus as a vector system allowed for straightforward transduction of a relatively high percentage of cells with integrated DNA resulting in sustained DsRed-uPAR transgene. The DsRed fluorescent marker allowed us to use cell sorting to further purify the population of cells capable of inducing transgene expression. In both cell lines transduced with the inducible uPAR virus, doxycycline stimulated substantial and dose-dependent uPAR and DsRed expression, while uninduced cells exhibited no leak-through of transgene expression. The induced uPAR was biologically active as demonstrated by modestly increased proliferation and markedly increased adhesion to vitronectin and resistance to anoikis. Since epithelial cancers have a high mortality within the circulation [10] due in large measure to apoptosis induced by matrix detachment, uPAR induction might help ensure survival during hematogenous or lymphatic metastasis stages.

Most previous evidence of uPAR's role in preventing apoptosis have relied upon experimentally reduced uPAR using techniques such as RNAi [11-13] rather than over-expression of the uPAR protein. Using the RNAi technique, Alfano et al [11] recently inferred a role for uPAR in anoikis by reporting knock-down of uPAR increased anoikis in nontransformed retinal epithelial cells. We directly demonstrated that increasing uPAR can confer resistance to anoikis in cancer cells using transient over-expression [4]. This present report demonstrates that inducement of stable uPAR results in anoikis resistance that is long lasting and extends to the LNCaP cell line; a cell line normally devoid of uPAR as well as to a PC-3M variant with little or no constitutive uPAR protein expression.

How uPAR promotes survival in suspension remains to be deciphered. This receptor has numerous reported associations with extracellular molecules [2]. uPAR could interact with these molecules to different degrees according to the environmental context of the cancer cell. For example, uPAR linkage with a transmembrane cell-surface receptor could increase during suspension. Extracellular matrix adhesion on the other hand might shift the balance of uPAR associations towards vitronectin and/or integrin binding. In this regard, the absence of association with matrix components and resulting loss of uPAR-matrix signaling might be as important to uPAR-induced resistance to anoikis as the presence of matrix components are to adhesion.

## Conclusion

Since the activities of uPAR play a critical role in several aspects of tumor progression and particularly in metastasis, inhibitors of uPAR which effectively restore anoikis may be of potential therapeutics benefit. Although no experimental therapies have yet attempted to target anoikis, more generalized inhibition of uPAR expression and/or activity in experimental mouse prostate cancer models have been shown to reduce occult metastasis [14], experimental bone metastasis [15], and cancer cell survival [9]. With models such as this inducible system, *in vivo *studies may now be designed to understand how upregulation of uPAR promotes discreet stages of metastasis. This information in turn may facilitate development of uPAR inhibitors that target specific roles of uPAR during metastasis as well as further define the role of this receptor in metastasis biology.

## Methods

### Cell culture

PC3M cells (a gracious gift from Dr. Isiah Fidler, MD Anderson Cancer Center, Houston TX) were maintained in Minimal Essential Medium with Earle's salts (MEM), supplemented with 10% fetal bovine serum, 100 units/mL penicillin, 100 μg/ml streptomycin, and 2.0 mM L-glutamine. A variant of the PC-3M line expressing low uPAR spontaneously arouse after serial passaging of the PC-3M; this variant is named PC-3M-CBS. We used PC-3M-CBS cells in this study. LNCaP cells were obtained from the American Type Culture Collection and maintained in RPMI medium (phenol-red free) supplemented with 10% fetal bovine serum, 100 units/mL penicillin and 100 μg/ml streptomycin sulfate. It was necessary to plate LNCaP cells on a poly-lysine surface during and after viral transduction to prevent high levels of cell detachment associated with transduction.

### Plasmids constructs and transduction

The 1.0 kb coding region of human uPAR was amplified using PCR from a pT7TSuPAR plasmid (a gift from Dr. K. Karico, U. Pennsylvania [16]) then cloned downstream from the carboxyl terminus of the DsRed monomer coding sequence in pDsRed-monomer-C1 vector (BD Biosciences). The inducible DsRed uPAR viral plasmid was constructed by cloning the DsRed-uPAR fusion cDNA into the pNL-TRE Pitt-EGFPΔU3 plasmid in place of EGFP [7] to generate pNL-TRE Pitt-DsRed-uPAR ΔU3. Virus was generated by transfecting 293 T cells with pNL-TRE Pitt-DsRed-uPAR ΔU3 plus pBHAΔΔΔ viral helper plasmid and pLTRVSVG viral envelop protein plasmid in a ratio of 3 parts DsRed-uPAR transgene, 2 parts helper and 1 part envelop. The calcium phosphate precipitation was used. Supernatant virus from 293T conditioned media was collected, filtered through 0.2 um membrane and concentrated by ultracentrifugation. This concentrated NL-TRE Pitt-DsRed-uPAR ΔU3 viral stock was then used to transduce prostate cancer cells.

PC-3M-CBS and LNCaP cells were transduced using both NL-TRE Pitt-DsRed-uPAR ΔU3 and NL-rtTA-M2 virus to create an inducible lentiviral delivered system. To induce expression of the DsRed-uPAR fusion, cells were stimulated with doxycycline HCL.

To control for any effects related to DsRed expression, a control DsRed-only virus was generated by cloning the DsRed coding sequence into the viral plasmid pNL-EGFP/CMV-WPRE(delta3) in place of the EGFP gene. The virus generated by this plasmid (NL-DsRed control/CMV-WPRE(delta3) produces constitutive DsRed expression in transduced cells.

### Western Blotting

Western Blot analysis for uPAR has been previously described [8]. Antibodies used for experiments in this study were: mouse anti-human uPAR (MAB 807) from R&D Systems, rabbit anti-DsRed (6224499) from Clontech and mouse anti-human beta actin (ab4321) AbCam antibodies.

### Immunofluorescence

Cells were fixed and permeabilized for immunofluorescence as we have described previously [4]. To detect uPAR, a primary antibody (anti-uPAR monoclonal, R&D Systems MAB 807) was then applied to cells for 1.5 hours followed by addition of AlexaFluor 488-tagged secondary antibody (Molecular Probes Inc.). Epifluorescence was imaged through a Zeiss Axiovert 200 fluorescent microscope and recorded through a Q-Imaging Retiga 2000R digital camera. Because uninduced cells expressed little or no fluorescence, phase contrast images of cells in the field of view were also captured to demonstrate relative position and number of these cells.

### Flow cytometry

Prostate cancer cells from viral-transduced adherent cultures were left uninduced or were induced with doxycycline for 4 days and then stained for uPAR as described [4]. Cells were next fixed in 1.0% paraformaldahyde/2.0% goat serum in PBS and analyzed by flow cytometry for both DsRed and uPAR expression.

### Proliferation assay

Cancer cells were seeded at 10,000 cells per well into 24 well plates and allowed to attach overnight. Human recombinant urokinase (100 ng/ml) was added to treatment wells in stock medium but with reduced levels of fetal bovine serum (2%). Quadruplicate wells were counted per condition on days 2, 4 and 6 of growth using a hemocytometer.

### Vitronectin adhesion assay

Twenty-four well tissue culture plates were coated overnight with 3.0 ug/well of human vitronectin (purchased from Sigma Chemicals). The coated wells were washed twice with PBS then blocked with 1 ml of 1% BSA in PBS for 1 hour at 37°C. Following a second PBS wash, 1.5 × 10^5 ^PC-3M-CBS and LNCaP cells suspended in stock culture medium plus 100 pM uPA were seeded onto the matrices or into BSA only-blocked wells (for a background measurement) for 1 hour at 37°C. Non-adherent cells were removed by aspiration and all wells were then washed twice with PBS. Cells adhering to the vitronectin surface were removed by trypsinization and counted. Results are the mean and standard deviation of 4 wells for each cell line.

### Assay to determine anoikis

To initiate apoptosis as a result of detachment (anoikis), doxycycline-induced, uninduced, DsRed-expressing controls and wild-type cells were removed from culture surfaces with 0.4% EDTA in PBS, washed to remove EDTA and resuspended in growth medium supplemented with heat-inactivated FBS to a final concentration of 500,000 cells in 5.0 mL. This cell suspension was placed in a 50-mL polypropylene conical tube coated with poly-HEMA (poly (2-hydroxyethyl methacrylate; (Sigma-Aldrich chemicals) to prevent re-attachment [17,18]. Aliquots of the cell suspension were analyzed each day to determine an overall percentage of anoikis for each cell condition.

To quantitate numbers of prostate cancer cells which became committed to apoptotic pathways, cell suspensions were stained using two dyes: Calcein Blue AM (Molecular Probes) and Annexin V-Alexa 488 (Molecular Probes) for 2 hours at 37°C. The Calcein dye is an esterified probe identifying live cells as it penetrates live cells and is then hydrolyzed freeing the fluorescent Calcein blue (excitation peak 373 nm, emission peak 440 nm). Annexin V staining identifies an early stage during the apoptotic process as it binds to phosphatidylserine residues inverted on the plasma membrane. Cell samples were then viewed using epifluorescence and 9 fields were scored as either 1) apoptotic (green fluorescence) or non-apoptotic (blue and not green). Anoikis measurements were carried out for 6 days in PC-3M-CBS cells while measurements were halted after 5 days in LNCaP cells because wild-type, DsRed control and uninduced apoptotic levels reached approximately 90%.

## Abbreviations

uPAR urokinase receptor

uPA urokinase-type plasminogen activator enzyme

## Competing interests

The author(s) declare that they have no competing interests.

## Authors' contributions

IS performed some experimental procedures, designed the study and drafted the manuscript. MH and RK designed and constructed vectors and lentivirus. RK and JR sorted the cells. JR participated in the development and design.
